# Assessment of salt intake to consider salt as a fortification vehicle for thiamine in Cambodia

**DOI:** 10.1111/nyas.14562

**Published:** 2021-01-07

**Authors:** Kathleen Chan, Jelisa Gallant, Shalem Leemaqz, Dare A. Baldwin, Mam Borath, Hou Kroeun, Jeffrey R. Measelle, Rem Ngik, Sophonneary Prak, Frank T. Wieringa, Lisa N. Yelland, Tim J. Green, Kyly C. Whitfield

**Affiliations:** ^1^ Department of Applied Human Nutrition Mount Saint Vincent University Halifax Nova Scotia Canada; ^2^ Women and Kids Theme South Australian Health and Medical Research Institute Adelaide South Australia Australia; ^3^ Department of Psychology University of Oregon Eugene Oregon; ^4^ National Sub‐Committee for Food Fortification Cambodia Ministry of Planning Phnom Penh Cambodia; ^5^ Cambodia Country Office Helen Keller International, Cambodia Phnom Penh Cambodia; ^6^ National Nutrition Programme Cambodia Ministry of Health Phnom Penh Cambodia; ^7^ UMR‐204 NutriPass Institut de recherche pour le développement Montpellier France; ^8^ School of Public Health The University of Adelaide Adelaide South Australia Australia; ^9^ School of Medicine The University of Adelaide Adelaide South Australia Australia

**Keywords:** salt, fortification, thiamine, beriberi, human milk, urinary sodium

## Abstract

Thiamine deficiency is a public health issue in Cambodia. Thiamine fortification of salt has been proposed; however, the salt intake of lactating women, the target population, is currently unknown. We estimated salt intakes among lactating women (<6 months postpartum) using three methods: repeat observed‐weighed intake records and 24‐h urinary sodium excretions (*n = *104), and household salt disappearance (*n = *331). Usual salt intake was estimated by adjusting for intraindividual intakes using the National Cancer Institute method, and a thiamine salt fortification scenario was modeled using a modified estimated average requirement (EAR) cut‐point method. Unadjusted salt intake from observed intakes was 9.3 (8.3–10.3) g/day, which was not different from estimated salt intake from urinary sodium excretions, 9.0 (8.4–9.7) g/day (*P* = 0.3). Estimated salt use from household salt disappearance was 11.3 (10.7–11.9) g/person/day. Usual (adjusted) salt intake from all sources was 7.7 (7.4–8.0) g/day. Assuming no stability losses, a modeled fortification dose of 275 mg thiamine/kg salt could increase thiamine intakes from fortified salt to 2.1 (2.0–2.2) mg/day, with even low salt consumers reaching the EAR of 1.2 mg/day from fortified salt alone. These findings, in conjunction with future sensory and stability research, can inform a potential salt fortification program in Cambodia.

## Graphical abstract

Thiamine deficiency is a public health issue in Cambodia. Thiamine fortification of salt has been proposed; however, the salt intake of lactating women is currently unknown. We estimated salt intakes among lactating women using three methods. Based on these data, a thiamine salt fortification scenario was modeled, which allowed the calculation of a suitable fortification dose of thiamine to add to salt.

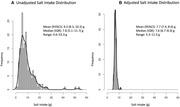

## Introduction

Thiamine deficiency is a persistent public health problem in Cambodia,^1^, ^2^, ^3^, ^4^ attributable to a diet consisting primarily of polished white rice and fish.^5^, ^6^ This deficiency can result in a range of cardiovascular and neurological conditions known collectively as thiamine deficiency disorders (TDDs).^4^ Of gravest concern is deficiency among infants, as TDDs can lead to irreversible cognitive deficits^7^ or often fatal infantile beriberi.^8^ To prevent infantile thiamine deficiency, maternal status must be improved as human milk thiamine concentrations are dependent on maternal intake.^9^

Thiamine has a short half‐life, and, therefore, must be consumed regularly to maintain adequate status.^10^ Mandatory fortification is a cost‐effective, sustainable, and passive means of increasing micronutrient intakes in a population.^11^ Thiamine fortification programs^12^ have been successfully implemented in many countries; however, none exist in Cambodia. Two recent reviews of thiamine deficiency globally and in low‐ and middle‐income countries both recommend food fortification as an ideal intervention to address thiamine deficiency and prevent infantile beriberi at the population level.^4^, ^13^ Previous efficacy trials of thiamine‐fortified fish sauce were successful in improving the thiamine status of women, children, and breastfed infants.^14^, ^15^ However, commercially produced fish sauce may not reach the most vulnerable who may home‐make their own sauce, and it is not commonly consumed in other global regions where thiamine intake is of concern.^4^ Instead, salt has been proposed as a fortification vehicle for thiamine in Cambodia and other at‐risk countries^4^ as it is used everywhere and is consumed by all segments of the population, regardless of socioeconomic status or season.^16^

The development of a food fortification program requires detailed data on the consumption of the proposed fortification vehicle for appropriate micronutrient dosing.^11^ These efforts should first be focused on the target population: lactating women for thiamine in Cambodia. There are limited data available on salt intake in Cambodia; therefore, the purpose of this study was to assess salt intake among rural lactating women in order to model a fortification scenario of thiamine in salt. Specifically, we assessed salt intakes using three methods: household salt disappearance, repeat 12‐h observed‐weighed salt and condiment intake records, and repeat 24‐h urinary sodium excretions.

## Materials and methods

Various methods were employed to estimate salt intake. Salt use was estimated at the household level in the Household Salt Disappearance Study (Study 1), and salt intake was estimated among lactating women using observed‐weighed intake records and urinary sodium excretions in the Maternal Salt and Sodium Study (Study 2). All participants were taking part in a randomized controlled supplementation trial with the primary aim of determining the daily dose of thiamine required by lactating Cambodian women to optimize milk thiamine concentrations (clinicaltrials.gov entry NCT03616288).^17^ Full study details are published elsewhere,^17^ but briefly, participants were lactating mothers of infants <6 months old residing in rural areas of Kampong Thom, Cambodia. Ethical approval for this study was obtained from the National Ethics Committee for Health Research in Cambodia (#112/250NECHR), and the Mount Saint Vincent University Research Ethics Board in Canada (MSVU UREB #2017‐141).

### Sociodemographic characteristics

Household characteristics and sociodemographic data were collected at 2 weeks postpartum, as part of the larger trial.^17^ Data collected include education, daily per capita income, National Wealth Equity Index scores, household size, and maternal age. Education is reported as the highest education level attained by either the mother or father in the household. Daily per capita income was calculated from monthly income and household size, and then categorized based on the World Bank Lower Middle Income Class Poverty Line of $3.20 USD/day.^18^ Households were categorized into the National Wealth Equity Index quintile using the EquityTool survey, a standardized tool based on data from the 2014 Cambodian Demographic and Health Survey.^19^ Household size was determined as all members ≥2 years old living and regularly consuming meals in the home, and maternal age was determined at 2 weeks postpartum.

### Study 1: Household salt disappearance study

Household salt disappearance was assessed via a longitudinal, exploratory study from 2 through 24 weeks postpartum. At 2 weeks postpartum, all salt was removed from the home and replaced with study‐provided iodized salt in a standardized container, and the initial weight of the container was recorded (Tanita Corporation; scales sensitive to 1 gram). During fortnightly home visits, trained field officers weighed the salt containers, refilled and reweighed containers, and collected information about the number of people residing and eating in the household and various salt use practices during the previous 14 days. Households that withdrew from the larger trial before 6 weeks postpartum were excluded from analysis.

Daily salt disappearance (in grams) was computed for household members ≥2 years of age by dividing fortnightly salt disappearance by the number of household members (≥2 years) as well as the number of days in the observation period. These data are presented as the mean (95% CI) and median (IQR) over the 22‐week study period. Data were not normally distributed (Shapiro–Wilk test *P* < 0.05), therefore, comparisons were made using Mann–Whitney *U* tests (lean versus peak agricultural seasons) and Kruskal–Wallis tests with Dunn–Bonferroni post‐hoc analysis, when applicable (household size and National Wealth Equity Index quintile), with a statistical significance level of *P* < 0.05.

### Study 2: Maternal salt and sodium study

Salt and sodium intakes were measured in a subset of 104 women. We measured 12‐h observed‐weighed salt and condiment records, and 24‐h urinary sodium excretions. Sodium‐containing condiments of interest included table salt, fish sauce, soy sauce, monosodium glutamate (MSG), and *prahok*, a commonly consumed fermented fish paste.^20^ Data were collected from each woman on 2 nonconsecutive days within a 7‐day window between 8 and 22 weeks postpartum.

#### Repeat 12‐h observed‐weighed salt and condiment intake records

Weighed intake records are considered the most accurate and precise method of intake assessment, and observed‐weighed intake records conducted by a trained field officer are recommended in low‐resource settings where literacy rates may be low.^21^ Twelve‐hour weighed intakes were collected from each woman by a trained field officer on both data collection days. Net weights of meals and snacks prepared, as well as salt and condiment use, were determined by weighing cooking pots and condiment containers before and after food preparation (Amir Technology Co. Ltd; scales sensitive to 0.1 gram). Maternal intake was estimated by weighing the dishes before and after consumption. Net discretionary salt and condiment use at mealtime was also recorded. Meals were usually prepared fresh each day, as refrigeration is not common in rural Cambodian households; however, in the rare instance that foods were consumed, but not prepared, during the observation period (i.e., leftovers), field officers recorded the net weight of the meal consumed and meal duplicates were developed at the end of the study to estimate intake.

Descriptive statistics (mean (95% CI) and median (IQR)) were computed for daily unadjusted intakes of salt and condiments. Sodium intakes from soy sauce, fish sauce, and *prahok* were calculated using the Association of Southeast Asian Nations (ASEAN) Food Composition Database Electronic Version 1,^22^ while sodium intakes from table salt and MSG were estimated using chemical composition. Salt (NaCl) intake from soy sauce, fish sauce, and *prahok* was estimated by converting sodium content to salt (in grams). Total estimated salt from all sources was computed by summing salt intake from table salt, soy sauce, fish sauce, and *prahok*. In this study, coverage is defined as consumption of the food item at least once during the observation day.

#### Twenty‐four‐hour urinary sodium excretions

The gold standard for assessing population sodium intake are repeat 24‐h urine collections, although this method cannot distinguish sources of sodium in the diet.^23^ Field officers conducted home visits the day before data collection to provide verbal instructions on the 24‐h urine collection procedure and to deliver urine collection materials. Participants were instructed to void their bladder the following morning and discard as usual, and then collect all urine voided up to and including the first void of the next morning.^24^ Field officers collected these urine samples within 24 h of the final void, weighed the sample, and then mixed it thoroughly before collecting 2‐mL aliquots for analysis. Samples were stored at −20 °C for ≤2 weeks before sodium measurement using ion‐selective potentiometry (EasyLyte Na/K/Cl Analyser, Medica). Urine samples <400 mL were considered incomplete and excluded from analysis.^25^ Mean (95% CI) and median (IQR) urine sample volumes and unadjusted 24‐h urinary sodium excretions were computed. Estimated salt intake from urinary sodium was calculated by assuming all sodium consumed was from salt (NaCl).

### Thiamine fortification modeling

A potential thiamine‐fortified salt scenario was created by modeling the usual salt intake distribution among lactating women, then determining a salt fortification dose via a modified estimated average requirement (EAR) cut‐point method using the EAR (1.2 mg thiamine/day) as a supplementation dose.^26^ This model does not control for the stability of a thiamine‐fortified salt, such as possible thiamine losses related to storage conditions or through cooking.

#### Usual salt intake distribution

Dietary intake data must be adjusted for intraindividual variation (i.e., usual intake) to be used in fortification modeling, as usual intake is more representative of the long‐term average intake in a population.^27^, ^28^ For this analysis, the National Cancer Institute (NCI) method was used to adjust for intraindividual variation.^27^, ^29^ The NCI method involves a one‐parameter Box–Cox transformation, followed by a linear mixed effects model to estimate adjusted individual intakes, and back‐transformation to the original scale. The adjusted estimates can then be used to plot the distribution of usual intakes in the population.^29^ In this study, usual daily salt intake from all major sources (table salt, fish sauce, soy sauce, and *prahok*) was estimated with the NCI method using data from the repeat 12‐h observed‐weighed salt and condiment intake records. This was decided *a priori* rather than 24‐h urinary sodium excretions, as MSG is a known source of sodium in the diet and would be indistinguishable from sodium from salt in urine samples.

#### Fortification scenario with modified EAR cut‐point method

The EAR cut‐point method is a common approach to setting fortification targets.^11^ With this method, intakes from a fortified food are modeled to minimize the proportion of micronutrient intakes from all sources below the EAR and above the tolerable upper intake level (UL).^30^ Here, a modified approach was employed, using a supplemental dose at the EAR level (1.2 mg) instead of assessing intake from all sources, and since thiamine does not have a UL,^26^ an upper parameter of 10 mg (a known perinatal supplemental dose currently used in Myanmar)^4^ was used in lieu of a UL. Iterative models were run with different fortification concentrations (mg thiamine/kg salt) in an attempt to minimize the proportion of thiamine intakes from salt below 1.2 mg/day or above 10 mg/day.

## Results

### Participants

**Figure 1:**
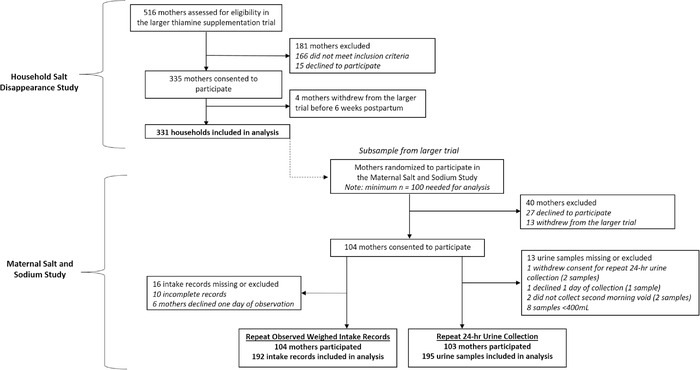
Participant flow chart for the Household Salt Disappearance Study and the Maternal Salt and Sodium Study.

A total of 331 households were included in the Household Salt Disappearance Study, and 104 women were included in the Maternal Salt and Sodium Study (Fig. 1). Most households had between three and six members (73%) and daily per capita incomes below the Cambodian poverty line (86%; Table 1). The subsample of mothers who participated in the Maternal Salt and Sodium Study had similar characteristics to those who were only in the Household Salt Disappearance Study (*P* > 0.05 for all sociodemographic characteristics).

**Table 1:** Household and maternal sociodemographic characteristics of study participants

	Household Salt Disappearance Study	Maternal Salt and Sodium Study		
Characteristic	All households (*n = *331)	Mothers included*^a^* (*n = *104)	Mothers not included (*n = *227)	*P* *^b^*
Mother's age (years)	28 (27–29)	28 (27–29)	28 (27–29)	0.89
Household size*^c^*				0.77
≤2 persons	27 (8%)	10 (10%)	17 (8%)	
3−6 persons	242 (73%)	74 (71%)	168 (73%)	
≥7 persons	62 (19%)	20 (19%)	42 (19%)	
Daily per capita income*^d^*				0.39
≤$3.20 USD	284 (86%)	91 (88%)	193 (85%)	
>$3.20 USD	47 (14%)	13 (12%)	34 (15%)	
Household education level*^e^*				0.77
None	10 (3%)	2 (2%)	8 (4%)	
Primary school	125 (38%)	37 (36%)	88 (39%)	
Lower secondary school	122 (37%)	38 (37%)	84 (37%)	
Upper secondary school	56 (17%)	21 (20%)	35 (15%)	
Higher education	18 (5%)	6 (5%)	12 (5%)	
National Wealth Equity Index quintile*^f^*				0.76
Lowest	80 (24%)	23 (22%)	57 (25%)	
Second lowest	69 (21%)	21 (20%)	48 (21%)	
Middle	107 (32%)	35 (34%)	72 (32%)	
Second highest	53 (16%)	15 (14%)	38 (17%)	
Highest	22 (7%)	10 (10%)	12 (5%)	

Note: Data are presented as *n* (%) or mean (95% CI).

^*a*^ Participants in the Maternal Salt and Sodium Study were randomly selected from the Household Salt Disappearance study.

^*b*^ ***χ*^2^** test or Mann–Whitney *U* test, comparing subsample of mothers (*n = *104) with remaining larger sample (*n = *227).

^*c*^ Includes household members ≥2 years of age.

^*d*^ Daily per capita income categorized as below or above the World Bank Lower Middle Poverty Line ($3.20 USD/person/day).

^*e*^ Household education level is defined as the highest education level attained within the household (either mother or father).

^*f*^ National Wealth Equity Index score is calculated using EquityTool, quintiles standardized to the 2014 Cambodian Demographic and Health Survey^19^ (https://www.equitytool.org/cambodia/).

### Study 1: Household salt disappearance study

As shown in Table 2, mean (95% CI) household salt use throughout the 22‐week study period was 11.3 (10.7–11.9) g/person/day, with more salt used during the lean (April–May) than the peak (November–December; *P* < 0.01) agricultural season. Households rarely reported selling, sharing, or using the study‐provided salt for business purposes, although salt was used to clean fish and/or vegetables in 27% (*n = *666) of fortnightly visits (*n = *181 households; data not shown).

**Table 2:** Fortnightly salt disappearance (g) among lactating women's households in rural Cambodia between 2 and 24 weeks postpartum

	*n* (households)	Mean (95% CI)	Median (IQR)	*P* *^a^*
Daily salt use/person/day	331	11.3 (10.7−11.9)	9.6 (7.2−13.5)	–
Household size*^b^*				0.05
≤2 persons	27	11.0 (8.3−13.7)	10.1 (6.6−13.8)	
3−6 persons	242	11.6 (10.9−12.3)	10.2 (7.4−13.8)	
≥7 persons	62	10.2 (8.7−11.7)	8.9 (6.3−11.6)	
National Wealth Equity Index quintile*^c^*				0.02
Lowest	81	11.6 (10.3−12.9)	10.1 (7.3−15.0)^1^	
Second lowest	69	11.4 (10.2−12.6)	10.2 (7.5−13.4)^1^	
Middle	106	12.1 (10.8−13.3)	10.7 (7.9−14.0)^1^	
Second highest	53	10.5 (9.1−11.9)	9.0 (6.9−13.5)^1^, ^2^	
Highest	22	8.0 (6.6−9.5)	7.3 (5.7−10.6)^2^	
Agricultural season	*n* (observations*^d^*)			<0.01
Lean season (April*–*May)	396	12.4 (11.6−13.4)	10.2 (6.3−16.9)	
Peak season (November*–*December)	1030	10.5 (10.0−10.9)	8.4 (5.5−12.6)	

^*a*^ Assessment for differences in salt disappearance by household size and National Wealth Equity Index quintile were evaluated using the Kruskal–Wallis test (with the Dunn–Bonferroni post‐hoc test; different superscript numbers in the same column indicate significant differences); differences by agricultural season were assessed using the Mann–Whitney *U* test.

^*b*^ Includes household members ≥2 years of age.

^*c*^ National Wealth Equity Index score is calculated using EquityTool, quintiles standardized to the 2014 Cambodian Demographic and Health Survey^19^ (https://www.equitytool.org/cambodia/).

^*d*^ Observation is defined as one fortnightly home visit.

### Study 2: Maternal salt and sodium study

Unadjusted results from the repeat 12‐h observed‐weighed salt and condiment intake records are shown in Table 3. Estimated total daily salt intake from table salt, soy sauce, fish sauce, and *prahok* was 9.3 (8.3–10.3) g/day, with table salt being the major contributor.

**Table 3:** Unadjusted repeat salt and sodium intakes from 12‐h observed weighed intake records (*n = *104 participants)

		Observed weighed intake (g/day)		Sodium intake*^a^* (g/day)	
	*n* (observations*b* *^,^* *c*)	Mean (95% CI)	Median (IQR)	Mean (95% CI)	Median (IQR)
Table salt	192	6.4 (5.6−7.1)	5.1 (3.2−7.9)	2.51 (2.21−2.81)	2.01 (1.27−3.11)
Soy sauce	192	0.2 (0.0−0.4)	0 (0−0)	0.01 (0−0.02)	0 (0−0)
Fish sauce	192	9.9 (7.4−12.3)	5.5 (1.1−12.0)	0.92 (0.69−1.14)	0.51 (0.10−1.12)
Monosodium glutamate (MSG)	144	3.6 (2.9−4.2)	2.8 (1.7−4.1)	0.44 (0.36−0.53)	0.34 (0.20– 0.50)
*Prahok* *^d^*	115	5.6 (3.3−7.8)	1.5 (0.0−8.1)	0.35 (0.21−0.49)	0.09 (0.00−0.51)
**Total salt** *^**e**^* **and sodium intakes**	**192**	**9.3 (8.3−10.3)**	**7.8 (5.1‐11.7)**	**3.61 (3.18−4.04)**	**3.15 (2.18−4.73)**

^*a*^ Sodium content of soy sauce, fish sauce, and *prahok* was calculated using the ASEAN Food Composition Database.^22^ Sodium content of table salt and MSG was computed based on chemical composition.

^*b*^ Observation is defined here as one 12‐h period (sunrise to sunset) of observed weighed salt and condiment intakes; missing *n = *16 observations due to incomplete records (*n = *10) and declined participation for 1 day of observation (*n = *6).

^*c*^ *n* differs due to changes in questionnaire (addition of MSG and *prahok*) to reflect field observations.

^*d*^ *Prahok* is a fermented fish paste condiment containing salt.

^*e*^ Total salt includes table salt, soy sauce, fish sauce, and *prahok*; calculation assumes sodium content from soy sauce, fish sauce, and *prahok* is from salt added during preparation/processing.

Based on urinary sodium, estimated daily salt intake was 9.0 (8.4–9.7) g/day (Table 4). Mean estimated total daily salt intake from observed‐weighed intake records and 24‐h urinary sodium excretions were not significantly different (*P* = 0.3; Mann–Whitney *U* test, data not shown).

**Table 4:** Unadjusted repeat 24‐h urinary sodium excretions and estimated salt intakes (*n = *103 participants)

	*n* (samples*a* *^,^* *b*)	Mean (95% CI)	Median (IQR)
Urine volume (L)	195	1.1 (1.0−1.2)	1.0 (0.7−1.4)
Urinary sodium excretion (g/24‐h)	195	3.6 (3.3−3.8)	3.2 (2.2−4.5)
Estimated salt intake*^c^* (g)	195	9.0 (8.4−9.7)	8.2 (5.6−11.4)

^*a*^ A sample is defined here as one 24‐h urine collection.

^*b*^ Missing *n = *13 samples due to incomplete collection (total 24‐h urine volume <400 mL, *n = *8), participant declined participation (*n = *3), or participant did not collect first void of the second morning (*n = *2).

^*c*^ Assumes all sodium consumed was from salt (NaCl) intake.

### Thiamine fortification modeling

**Figure 2:**
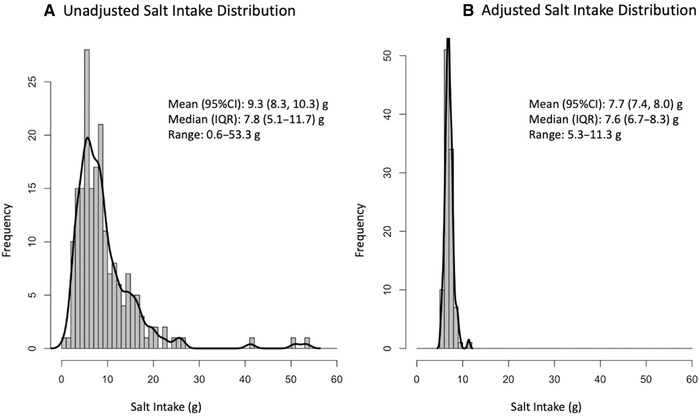
Salt intake distributions (from table salt, soy sauce, fish sauce, and *prahok*) for lactating women in rural Cambodian before (A) and after (B) adjusting for intraindividual variance using the National Cancer Institute Usual Dietary Intake method.^29^

After adjusting for intraindividual variance using the NCI method, mean usual salt intake from table salt only was 7.0 (6.9–7.2) g/day, while mean usual salt intake from all sources (table salt, soy sauce, fish sauce, and *prahok*) was 7.7 (7.4–8.0) g/day (distribution before and after adjustment in Fig. 2). Assuming no stability losses, a thiamine fortification dose of 275 mg thiamine/kg salt was determined to meet scenario parameters (minimize intakes below 1.2 mg/day and above 10 mg/day through fortified salt). At this level of fortification, mean thiamine intakes from fortified salt would be 2.1 (2.0–2.2) mg/day, ranging from 1.2 to 4.3 mg/day.

## Discussion

This was the first study to assess salt intake among lactating rural Cambodian women, who we estimate consume 9 g salt/day, with close agreement between estimates obtained via observed‐weighed intake records and urinary sodium excretions. When adjusted for usual intake, salt intake estimates reduce to 7–8 g/day. Mandatory food fortification is a cost‐effective and efficacious intervention to improve the micronutrient status of a population,^11^ and salt has been proposed as a fortification vehicle for thiamine in Cambodia.^4^ Our preliminary model suggests that salt fortified with 275 mg thiamine/kg salt could result in thiamine intakes from salt alone ranging from 1.2 to 4.3 mg/day. Future research on the stability and sensory characteristics, as well as analysis of cost and price implications of any thiamine‐iodine‐salt formulation, should be used to inform a thiamine fortification program in Cambodia.

### Salt and condiment intake in Cambodia

There has been limited investigation of salt and sodium intakes in Cambodia, with no disaggregated data specifically for lactating women previously available. In this study, both 12‐h observed‐weighed salt and condiment intake records and 24‐h urinary sodium gave similar salt estimates (9.3 (8.3–10.3) versus 9.0 (8.4–9.7) g/day, respectively). These results were not significantly different, which suggests that both observed‐weighed records and urinary sodium methods are appropriate measures of salt and sodium intake among lactating women in rural Cambodia, and only one assessment method is needed to reliably estimate salt and sodium intakes in this population. Our estimates are lower than previous estimates for salt intake; using food balance sheets, Powles *et al*. estimated mean sodium intakes of 4.14 (3.73–5.18) g/day, or approximately 11 g/day salt among Cambodian women,^31^ while Laillou *et al*. cited an average per capita salt consumption of 15 g/day for Cambodian adults.^32^ However, this is not unexpected, as food balance sheets describe salt production and import at the national level, but cannot account for waste or intake by population subgroups.^21^

Similarities between the estimated intake findings for these two methods also reveal that most sodium intake was captured in the observed‐weighed intake records, and thus the main source of sodium in the participants’ diets was from discretionary salt and condiment use. This is in line with previous research in the region^33^ and different than consumption in North America, where up to 70% of dietary sodium intake comes from commercially processed foods.^34^

### Salt as a thiamine fortification vehicle

The World Health Organization has indicated that salt fortification programs are compatible with the global salt reduction strategy aimed to reduce population salt intake to <5 g/person/day.^16^ As a passive intervention, mandatory fortification does not promote additional intake of the fortified food; instead, regular monitoring of usual salt intakes can ensure fortification levels that meet the needs of the population.^16^ Salt meets the criteria for an appropriate fortification vehicle, as it is consumed daily by participants, and in relatively consistent amounts. Another important consideration for this target population are postpartum food restrictions, which are common in several beriberi‐endemic countries in Southeast Asia; these diets do not appear to restrict salt.^35^

One unexpected finding was the limited use of soy sauce in rural Cambodian homes. Soy sauce was only used on 6% (*n = *11) of observation days, and only by 9 out of 104 participants (data not shown). This finding is notable given the considerable efforts to promote soy sauce as a fortification vehicle for iron in Cambodia.^36^ Iron‐fortified fish and soy sauce programs commenced in 2011, and by 2014, approximately 25% of domestically produced sauces were fortified.^37^ Given our findings, soy sauce may not be an appropriate fortification vehicle for any micronutrient in Cambodia if the intervention goal is to reach vulnerable, rural populations.

### Household salt disappearance and salt use practices

Fortnightly household salt disappearance was the least resource‐intensive assessment method employed, and while it was useful in understanding broader trends in salt use, this method overestimated table salt intake (11 g/person/day), could not provide intrahousehold intake patterns, and assessed only table salt rather than salt intake from all condiments. This method was helpful in uncovering the previously undocumented, yet common nonconsumptive practices of cleaning fish and vegetables with salt, which was reported in 26% of fortnightly visits. The frequency of this practice provides further context into the overestimate, and should be considered if this assessment method is used in rural Cambodia or similar settings in the future. In addition, salt use was found to be lower during fortnightly visits in the peak agricultural season as compared with the lean agricultural season. This finding should be explored further, as it is unclear if this difference is related to nonconsumption uses, such as cleaning fish and vegetables, for food preservation purposes in the early lean season, or if salt is being consumed in higher amounts during the lean season.

### Thiamine fortification of salt

We modeled a preliminary fortification scenario of thiamine‐fortified salt using adjusted salt intakes from the repeat 12‐h observed‐weighed salt and condiment intake records (7.7 (7.4–8.0) g/day) and the EAR level supplementation dose of thiamine for lactating women (1.2 mg/day).^26^ Using a modified EAR cut‐point method, a salt fortification dose of 275 mg thiamine/kg salt was modeled to increase thiamine intakes above the EAR level supplementation dose of thiamine for lactating women (1.2 mg/day), yet still remains well below a known safe supplemental dose of 10 mg/day.^4^ While the NCI method is known to minimize the 10th and 90th percentiles of intakes in modeled distributions,^38^ the highest intake of thiamine from fortified salt was well below the known safe dose (4.3 mg/day at the 99th percentile of intake), therefore, this fortification dose is likely still appropriate even if usual salt intake has been underestimated in this model.

This initial model does not consider several important technical aspects of thiamine‐fortified salt, such as potential storage‐related thiamine degradation in the salt matrix, which would inform a more robust fortification model. Further research is needed on the stability, organoleptic, and sensory characteristics of a thiamine‐fortified salt. While some micronutrients, such as iron, must be microencapsulated for various technological and sensory reasons, recent evidence indicates that spray‐fortification of salt with both folic acid and iodine is feasible, indicating the potential for other B vitamins.^39^ In addition, although thiamine hydrochloride is one of the least expensive fortificants, future research in this area is also warranted. Previous estimated costs of thiamine hydrochloride were $24 USD/kg, with an estimated cost of $0.018 USD/year to meet the EAR for an adult male through a fortified dry food.^11^ To put this in context, iron (NaFeDTA) had an estimated cost of $0.383 USD/year to meet the requirements of an adult male.^11^

### Sources of fortified salt in the diet

In Cambodia, salt is categorized as *coarse* or *refined*. Coarse salt is produced through solar evaporation, while refined salt undergoes additional boiling and processing to create a finer grain size.^32^ Both types of salt fall within iodization legislation in Cambodia, and may be consumed as table salt or used to produce food products, such as fish sauce or *prahok*.^40^ There has historically been greater adherence to iodization policies among refined salt producers; therefore, the source of salt consumed is an important consideration for salt fortification programs.^32^ There is some evidence that household use of refined salt has been increasing.^41^ The Ministry of Planning Cambodia and UNICEF collected salt samples from students’ households at 72 primary schools in 2008 (*n = *2329) and 2011 (*n = *2310) and found refined salt use increased from 42% to 53% over this time period.^42^, ^43^ There is a need for updated salt purchasing data, and this information should be considered as part of a thiamine‐fortified salt program.

The preliminary fortification model presented used usual salt intake from all major sources, as opposed to table salt only, to reflect the current Cambodian food manufacturing policies that require the use of fortified salt in processed foods.^40^ Although compliance to this legislation is variable throughout Southeast Asia, it has been satisfactory in Cambodia,^44^ where most table salt and salt‐containing condiments are produced locally.^36^ While this may not be the case for all countries, it is important to note that table salt intake was the largest and most consistently consumed salt source, while condiment intake was highly variable. In fact, the preliminary fortification dose of 275 mg thiamine/kg salt also met our fortification model parameters (minimizing intakes below 1.2 mg/day and above 10 mg/day) when table salt was modeled alone. It is possible that condiment intakes contributed mostly as a source of variance in salt intake, which was reduced when intakes were adjusted for intraindividual variance with the NCI method. This also suggests that the assessment of table salt intake alone may be sufficient for future monitoring of usual salt intake in this population.

### Strengths, limitations, and future directions

This study had several strengths: it was the first study to assess salt intake and model salt as a fortification vehicle for thiamine among lactating women in Cambodia, and it employed multiple methods of salt and sodium assessment. Some limitations for this study include a relatively small sample size for the Maternal Salt and Sodium Study. While the sample size (*n = *104) was sufficient to adjust for intraindividual variance, it was not large enough to explore additional intake trends such as changes by agricultural season. Another limitation was the use of the ASEAN Food Composition Database to estimate the sodium content of fish sauce, soy sauce, and *prahok*. This is standard practice for estimating the nutrient content of foods; however, the salt content in fish sauce and soy sauce is known to be variable in Cambodia.^37^

Future research should examine the costs and technological aspects of thiamine‐iodine–fortified salt, such as stability and acceptability testing under various storage and cooking conditions and after food processing (e.g., commercial production of sauces), as well as the efficacy of thiamine‐iodine–fortified salt to improve infantile thiamine status in Cambodia. Furthermore, an updated assessment of the salt content of condiments produced in Cambodia and current iodized salt production practices in food processing would provide insight into future opportunities and barriers to a national thiamine‐fortified salt program.

## Conclusions

Lactating Cambodian women consume approximately 9 g/day salt, and most salt in the diet comes from discretionary table salt used for cooking. Salt was consumed daily and in relatively consistent amounts, regardless of socioeconomic status, confirming that salt would be an appropriate fortification vehicle for thiamine in Cambodia. A mandatory thiamine fortification program of salt may help reduce the prevalence of thiamine deficiency in rural Cambodia and prevent TDDs among the most vulnerable segment of the population, exclusively breastfed infants. Future research should focus on the feasibility of such a program, including studies on the technical and cost‐related aspects of dual thiamine‐ and iodine‐fortified salt, efficacy trials, and quality control measures.

## Author contributions

K.C. and K.C.W. drafted the manuscript. K.C.W., H.K., T.J.G., F.T.W., J.R.M., and D.A.B. conceived the study and wrote the initial study protocol. M.B., S.P., L.N.Y., S.L., N.R., K.C., and J.G. assisted in developing the protocol. N.R., H.K., M.B., and S.P. facilitated implementation of the study. J.G. and K.C. were involved in study coordination. L.N.Y. and S.L. developed the statistical analysis plan, and K.C. and S.L. performed analysis. K.C.W. accepts responsibility for the integrity of the data analyzed. All authors participated in, read, and approved the final manuscript.

## Competing interests

The authors declare no competing interests.
